# Cross-national gender variations of digit ratio (2D:4D) correlate with life expectancy, suicide rate, and other causes of death

**DOI:** 10.1007/s00702-017-1815-7

**Published:** 2017-11-21

**Authors:** Bernd Lenz, Johannes Kornhuber

**Affiliations:** Department of Psychiatry and Psychotherapy, Friedrich-Alexander University Erlangen-Nürnberg (FAU), Schwabachanlage 6, 91054 Erlangen, Germany

**Keywords:** Digit ratio, Suicide, Causes of death, Life expectancy, Respiratory infections, Asthma

## Abstract

The second-to-fourth finger length ratio (2D:4D) is an indication of prenatal sex hormone exposure, and has sex-specifically been associated with several lethal illnesses including ischemic heart disease, diverse cancers, and suicide. Our primary aim was to verify that 2D:4D sex-specifically relates to life expectancy and suicide numbers on a national level (23 countries). We also used a hypothesis-free approach to investigate associations with other causes of death [*p* value adjustment for multiple hypothesis testing using the false discovery rate procedure (FDR)]. All parameters were normalized to the national mean (of males and females) and analyzed across nations. Normalized male 2D:4D correlated positively with normalized male life expectancy (at birth, *r* = 0.46, *p* = 0.029; at the age of 60, *r* = 0.44, *p* = 0.038) and negatively with normalized male suicide rates (*r* = − 0.49, *p* = 0.017). In the exploratory analyses, the normalized male 2D:4D values were negatively associated with the normalized male deaths rates from communicable, maternal, perinatal, and nutritional conditions [*r* = − 0.65, *p*(FDR) = 0.011], respiratory infections [*r* = − 0.69, *p*(FDR) = 0.008], asthma [*r* = − 0.65, *p*(FDR) = 0.011], neurological conditions [*r* = − 0.56, *p*(FDR) = 0.046], and Alzheimer’s disease and other dementias [r = − 0.59, *p*(FDR) = 0.036]. The normalized female parameters showed the same cross-national correlations. In line with the previous individual level findings, the results suggest that prenatal sex hormone effects are sex-specifically involved in suicide and neurological conditions. Moreover, we provide novel national level evidence that prenatal sex hormone priming may sex-specifically influence life expectancy and death risk from respiratory diseases.

## Introduction

Emerging evidence suggests that sex hormone levels during early developmental periods influence the risk of severe diseases with high mortality in later life. Based on preclinical causal and clinical associational studies, the second-to-fourth finger length ratio (2D:4D) is seen as a non-invasive retrospective biomarker for prenatal androgenization and estrogenization (Berenbaum et al. 2009; Breedlove 2010; Zheng and Cohn 2011; Auger et al. 2013) (but see also Wallen 2009 for a critical review). Many hypothesis-driven studies have shown associations between 2D: 4D and severe diseases. For example, lower (prenatally more androgenized) 2D:4D has been reported in individuals with poorer health (Rapoza 2017), prostate cancer (Mendes et al. 2016), primary brain tumors (Bunevicius et al. 2016), alcohol dependence (Kornhuber et al. 2011; Han et al. 2016; Lenz et al. 2017), anorexia nervosa in females (Quinton et al. 2011), Alzheimer’s disease in females (Vladeanu et al. 2014), and aggression-related injuries (Joyce et al. 2013; O’Briain et al. 2017). In addition, we recently provided the initial evidence for lower 2D:4D values in males who had died from suicide in comparison to males with other causes of death (Lenz et al. 2016). Higher (prenatally more estrogenized) 2D:4D has also been associated with lethal diseases such as breast cancer (Muller et al. 2012; Hong et al. 2014), cervical dysplasia (Brabin et al. 2008), oral squamous cell carcinoma in males (Nicolás Hopp and Jorge 2011), gastric cancer (Nicolás Hopp et al. 2013), disordered eating in males (Smith et al. 2010), Alzheimer’s disease in males (Vladeanu et al. 2014), coronary heart disease (Lu et al. 2015), and myocardial infarction (Kyriakidis et al. 2010). These studies, though, are often limited in sample size. Different methods for 2D:4D quantification complicate the comparison of investigations and demand cautious interpretation [e.g., Vernier caliper, photocopies, X-rays; usage of scans and photocopies results in lower 2D:4D in comparison to direct measures (Ribeiro et al. 2016)]. Most findings have not been replicated and thus negative or contradictory results have been reported [e.g., prostate cancer (Muller et al. 2011; García-Cruz et al. 2012)]. The overall effect sizes may be over interpreted due to publication bias of positive findings.

Of the above reported and 2D:4D-related diseases, ischemic heart disease and dementias reached the ranks of 1 (8.8 million deaths) and 7 (1.5 million deaths) for the World Health Organization (WHO) top 10 list of global causes of death in 2015 (WHO 2017). This indicates that prenatal sex hormone exposure may significantly contribute to the global death burden.

Our primary aims were to demonstrate a link between 2D:4D and life expectancy and to provide additional evidence for a role of 2D:4D in suicide. Moreover, we used a hypothesis-free approach to further explore associations with specific causes of death, as defined by WHO. Many studies have shown sex-dependent relationships between 2D:4D and illness with stronger or exclusively significant associations in males, suggesting that prenatal sex hormone priming might be more relevant to lifetime health status in males in comparison to females (Martin et al. 1999; Bailey and Hurd 2005; Martel et al. 2008; Collinson et al. 2010; Kyriakidis et al. 2010; Kornhuber et al. 2011; Nicolás Hopp et al. 2013; Portnoy et al. 2015; Lenz et al. 2016, 2017; Rapoza 2017) (but see also: Smedley et al. 2014; O’Briain et al. 2017). Thus, we analyzed sex-specifically whether normalized 2D:4D correlated with normalized life expectancy, suicide rates, and death rates from other causes across nations.

## Methods

We normalized the male and female variables of interest (2D:4D, life expectancies, suicide rates, death rates from specific causes) to their national means (= sex-specific value divided by the mean of the male and female values) and calculated cross-national Pearson correlations between normalized 2D:4D and normalized variables of interest for males and females. In comparison to the direct correlation of 2D:4D values with the variables of interest, the correlation of normalized 2D:4D with the other normalized variables of interest may reduce the risk of misleading associations induced by psycho-social confounders (e.g., national differences in health care systems).

We extracted the national 2D:4D values from a follow-up analysis of the BBC Internet study (Reimers 2007) investigating gender inequality between 29 nations (158, 753 participants) (Manning et al. 2014). Due to how ethnicity influences 2D:4D, predominantly Caucasian national samples were analyzed. An online self-measure method for length of the second and fourth fingers has been employed. To reduce the number of statistical tests, we used the means of the national right-hand and left-hand 2D:4D values. Although some studies show stronger associations between 2D:4D and the target traits for the right hand (e.g., Manning et al. 1998; Hönekopp and Watson 2010; Kornhuber et al. 2011; Masuya et al. 2015; Bilgic et al. 2016), others found stronger effects for the left hand (e.g., Muller et al. 2012; Kilduff et al. 2013; Kornhuber et al. 2013; Hong et al. 2014; Lu et al. 2015). To our knowledge, there is no reliable explanation for differing associations of right- and left-hand 2D:4D with prenatal androgen load.

For confirmatory testing, gender-specific national life expectancies (at birth and at the age of 60) and suicide rates (age-standardized suicide rates per 100,000) for the year 2012 were extracted from World Health Statistics (WHO 2014c) and the WHO suicide report (WHO 2014b). For exploratory analyses, point estimates for deaths (adjusted for the population size) by cause, sex, and Member State in 2012 from the Global Health Estimates summary tables were used (WHO 2014a). We included the following 23 countries, as they provided high-quality data for variables of interest [comprehensive vital registration with at least 5 years of data for suicide estimates (WHO 2014b) and direct nationally representative death registration data for death numbers (WHO 2014a)]: Australia, Austria, Belgium, Canada, Croatia, Czech Republic, Denmark, Finland, France, Germany, Hungary, Iceland, Ireland, Italy, Netherlands, New Zealand, Norway, Romania, Spain, Sweden, Switzerland, United Kingdom, and the United States of America.

Variables with a significant deviation from the normal distribution (Shapiro–Wilk test) were transformed into rankit normal scores (Bishara and Hittner 2012). The *p* values < 0.05 were considered statistically significant, and *p* values < 0.1 were interpreted as a trend. To manage type 1 error risk in exploratory analyses, we adjusted *p* values for multiple hypothesis testing with the false discovery rate (FDR) procedure using a macro for Microsoft Excel [Appendix S1 of Pike (2011), graphically sharpened method; (Benjamini and Hochberg 2000)]. Data were analyzed with SPSS for Windows 21.0 (SPSS Inc., Chicago, IL, USA) and Graph Pad Prism 5 (Graph Pad Software Inc., San Diego, CA, USA).

## Results

**Fig. 1:**
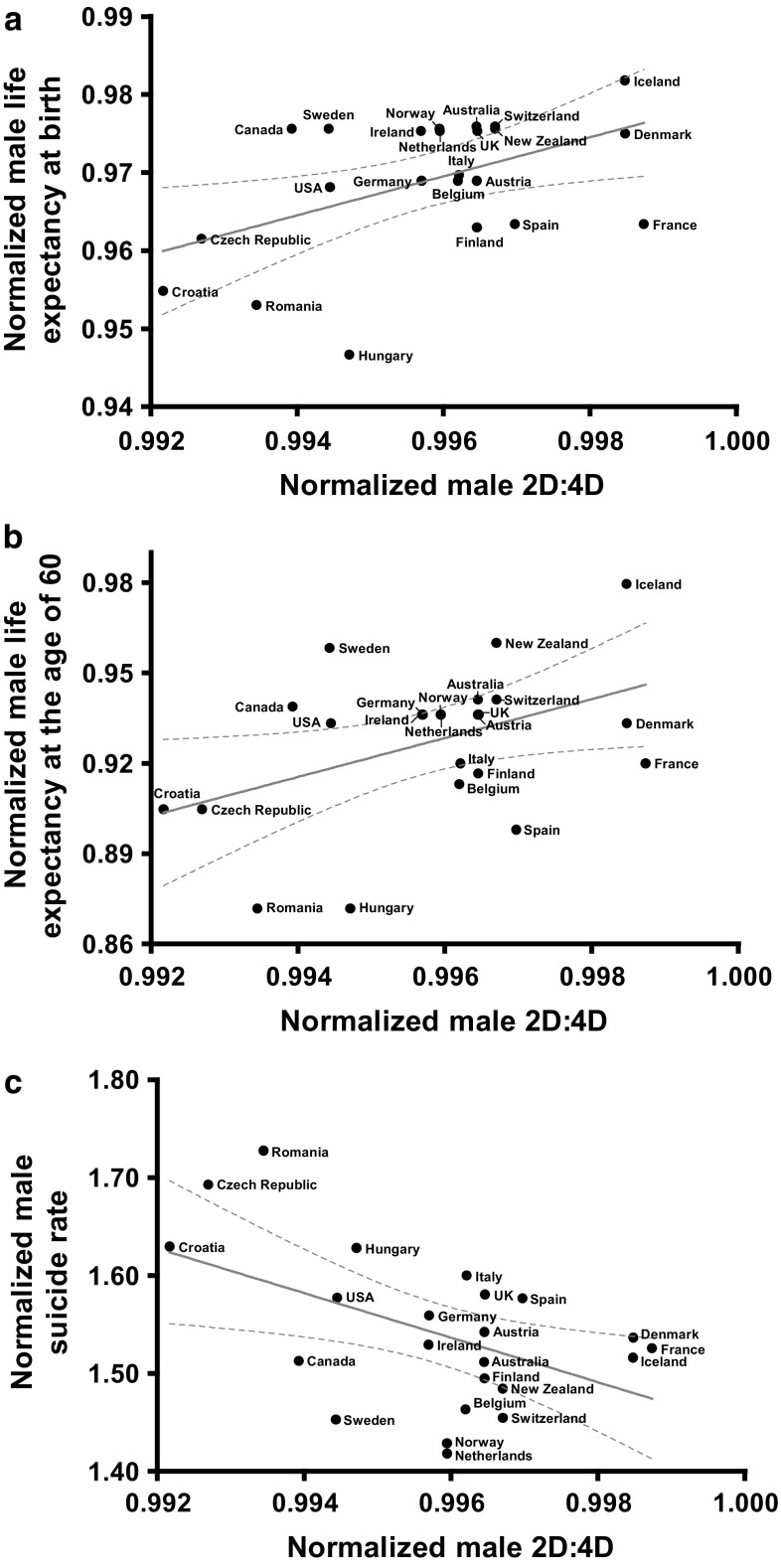
Normalized male 2D:4D values correlate positively with normalized male life expectancies at birth (*n* = 23, *r* = 0.46, *p* = 0.029; Fig. 1a) and at the age of 60 (*n* = 23, *r* = 0.44, *p* = 0.038; Fig. 1b) and negatively with normalized male age-standardized suicide rates (*n* = 23, *r* = − 0.49, *p* = 0.017; Fig. 1c). The analyzed variables were normalized to their national means (= male value divided by the mean of the male and female values), i.e., in countries with lower (prenatally more androgenized) national male 2D:4D values (divided by the national mean of the male and female values), we found lower male life expectancies at birth and at the age of 60 (divided by the national mean of the male and female values) and higher male age-standardized suicide rates (divided by the national mean of the male and female values). These cross-national associations indicate that lower 2D:4D ratios might sex-specifically be related to reduced life expectancy and increased risk for suicide at individual levels. For national sex-specific right- and left-hand 2D:4D values, see Table 1 in Manning et al. (2014); dotted lines represent the 95% confidence intervals of the best-fit from a linear regression analysis

In support of our confirmatory hypotheses, normalized male 2D:4D correlated positively with normalized male life expectancy (at birth, see Fig. 1a, at the age of 60, see Fig. 1b) and negatively with normalized age-standardized male suicide rates (see Fig. 1c) across nations. On an individual level, these findings indicate that lower 2D:4D values might sex-specifically be associated with reduced life expectancy and a higher risk for death by suicide.

Subsequently, we explored cross-national correlations between the normalized male 2D:4D and normalized male death rates from other specific causes, as defined by WHO (see Table 1). After correction for multiple hypothesis testing according to the FDR procedure, the analyses revealed exclusively negative Pearson correlations. Across nations, normalized male 2D:4D values were negatively related to the normalized male death rates of the following causes: Communicable, maternal, perinatal, and nutritional conditions; respiratory infections; neurological conditions; Alzheimer’s disease and other dementias; asthma; and other respiratory diseases. The normalized male 2D:4D values were only nominally related to death rates in the following groups: All causes, pancreas cancer, lymphomas and multiple myeloma, ischemic heart disease, digestive diseases, other digestive diseases, intentional injuries, and self-harm. For reasons of clarity and comprehensibility, we have focused on normalized male variables. However, we have repeated all analyses with normalized female variables of interest; the results showed the same correlations as in males (statistical values are not shown).

**Table 1:** Cross-national Pearson correlations between normalized male 2D:4D and normalized male death rates from specific causes [Global Health Estimates summary tables (WHO 2014a)]

	*r*	*p*	*p*(FDR)
All causes	− 0.43	**0.0391**	0.1485
I. Communicable, maternal, perinatal, and nutritional conditions	− 0.65	**0.0008**	**0.0109**
A. Infectious and parasitic diseases	− 0.36	0.0896	0.1929
1. Tuberculosis	− 0.39	0.0666	0.1769
4. Diarrheal diseases	0.12	0.6011	0.5271
6. Meningitis	− 0.09	0.6759	0.5504
7. Encephalitis	− 0.20	0.3698	0.4088
10. Parasitic and vector diseases	− 0.17	0.4319	0.4245
12. Other infectious diseases	− 0.26	0.2225	0.3381
B. Respiratory infections	− 0.69	**0.0003**	**0.0078**
1. Lower respiratory infections	− 0.69	**0.0003**	**0.0078**
D. Neonatal conditions	0.15	0.4960	0.4635
1. Preterm birth complications	0.31	0.1525	0.2484
4. Other neonatal conditions	− 0.25	0.2510	0.3460
E. Nutritional deficiencies	− 0.31	0.1485	0.2484
II. Non-communicable diseases	− 0.36	0.0948	0.1929
A. Malignant neoplasms	− 0.24	0.2608	0.3460
1. Mouth and oropharynx cancers	− 0.37	0.0782	0.1853
2. Esophagus cancer	− 0.39	0.0683	0.1769
3. Stomach cancer	0.19	0.3846	0.4136
4. Colon and rectum cancers	− 0.07	0.7574	0.5680
5. Liver cancer	− 0.07	0.7485	0.5680
6. Pancreas cancer	− 0.48	**0.0211**	0.1095
7. Trachea, bronchus, lung cancers	− 0.26	0.2333	0.3409
8. Melanoma and other skin cancers	− 0.02	0.9288	0.6618
14. Bladder cancer	− 0.03	0.8777	0.6333
15. Lymphomas, multiple myeloma	0.45	**0.0331**	0.1485
16. Leukemia	− 0.15	0.4851	0.4609
Other malignant neoplasms	− 0.18	0.4130	0.4240
B. Other neoplasms	− 0.36	0.0926	0.1929
C. Diabetes mellitus	− 0.05	0.8081	0.5905
D. Endocrine, blood, immune disorders	− 0.24	0.2640	0.3460
E. Mental and behavioral disorders	− 0.17	0.4289	0.4245
4. Alcohol use disorders	− 0.18	0.4166	0.4240
5. Drug use disorders	− 0.09	0.6986	0.5608
7. Eating disorders	− 0.21	0.3371	0.4003
11. Other mental and behavioral disorders	− 0.18	0.3993	0.4214
F. Neurological conditions	− 0.56	**0.0056**	**0.0456**
1. Alzheimer’s disease and other dementias	− 0.59	**0.0032**	**0.0361**
2. Parkinson’s disease	− 0.01	0.9768	0.6874
3. Epilepsy	− 0.29	0.1875	0.2968
4. Multiple sclerosis	− 0.25	0.2505	0.3460
7. Other neurological conditions	− 0.37	0.0787	0.1853
H. Cardiovascular diseases	− 0.11	0.6266	0.5412
1. Rheumatic heart disease	− 0.35	0.1019	0.2003
2. Hypertensive heart disease	− 0.40	0.0585	0.1769
3. Ischemic heart disease	0.44	**0.0361**	0.1485
4. Stroke	− 0.37	0.0813	0.1853
5. Cardiomyopathy, myocarditis, endocarditis	− 0.33	0.1183	0.2248
6. Other circulatory diseases	0.13	0.5481	0.5039
I. Respiratory diseases	− 0.40	0.0570	0.1769
1. Chronic obstructive pulmonary disease	− 0.10	0.6566	0.5485
2. Asthma	− 0.65	**0.0007**	**0.0109**
3. Other respiratory diseases	− 0.57	**0.0043**	**0.0413**
J. Digestive diseases	− 0.50	**0.0141**	0.0805
1. Peptic ulcer disease	− 0.41	0.0522	0.1769
2. Cirrhosis of the liver	0.08	0.7153	0.5663
3. Appendicitis	0.26	0.2254	0.3381
Other digestive diseases	− 0.44	**0.0367**	0.1485
K. Genitourinary diseases	− 0.10	0.6640	0.5485
1. Kidney diseases	0.12	0.5954	0.5271
3. Urolithiasis	− 0.40	0.0601	0.1769
4. Other genitourinary diseases	− 0.23	0.2846	0.3528
L. Skin diseases	− 0.22	0.3191	0.3871
M. Musculoskeletal diseases	0.10	0.6406	0.5450
Other musculoskeletal disorders	− 0.08	0.7271	0.5677
N. Congenital anomalies	− 0.19	0.3729	0.4088
4. Congenital heart anomalies	− 0.20	0.3560	0.4088
5. Other chromosomal anomalies	− 0.31	0.1483	0.2484
6. Other congenital anomalies	− 0.17	0.4425	0.4275
III. Injuries	− 0.39	0.0641	0.1769
A. Unintentional injuries	− 0.32	0.1316	0.2345
1. Road injury	− 0.07	0.7561	0.5680
2. Poisonings	− 0.33	0.1282	0.2345
3. Falls	− 0.23	0.2847	0.3528
4. Fire, heat, and hot substances	− 0.24	0.2671	0.3460
5. Drowning	0.12	0.5813	0.5259
7. Other unintentional injuries	− 0.20	0.3655	0.4088
B. Intentional injuries	− 0.53	**0.0090**	0.0639
1. Self-harm	− 0.52	**0.0109**	0.0690
2. Interpersonal violence	− 0.05	0.8049	0.5905

The analyzed variables were normalized to their national means (= male value divided by the mean of the male and female values). A negative cross-national correlation between normalized male 2D:4D and normalized male death rates from specific causes means that lower (prenatally more androgenized) national male 2D:4D values (divided by the national mean of the male and female values) correlate with a higher national risk for cause-specific death in males (divided by the national mean of the male and female values). The normalized female parameters showed the same correlations. We calculated only correlations for which male and female causes of death estimates according to the Global Health Estimates summary tables (WHO 2014a) in all 23 countries included in this study were available. The following categories have been excluded due to missing national values: I: A2-3, A5, A8-9, A10a-k, A11, B2-3, C, D2-3, E1-5; II: A9-13, E1-3, E6, E8-10, F5-6, G, K2, K5-6, M1-4, N1-3, O; III: A6, B3. For national sex-specific right- and left-hand 2D:4D values, see Table 1 in Manning et al. (2014). Significant results (*p* < 0.05) are illustrated in bold letters. FDR False discovery rate

## Discussion

This investigation shows that, across nations, normalized male 2D:4D values correlate significantly with normalized male life expectancies (at birth and at the age of 60), suicide rates, and other cause-specific death rates (using a hypothesis-free approach). The reported associations are also true for females. However, for reasons of clarity and comprehensibility, we focus on males when elaborating the relationships in the following discussion.

Across nations, normalized male 2D:4D values were positively associated with normalized male life expectancies at birth and at the age of 60. This means that lower (prenatally more androgenized) male 2D:4D values (in relation to the mean of male and female 2D:4D) were found in countries with lower male life expectancies (in relation to the male and female mean). This observation supports the assumption that prenatal sex hormone priming entails long-lasting, possibly lifelong effects on sex-specific death risk. The exploratory analyses revealed significant associations between normalized male 2D:4D and normalized male death rates in seven specific causes of death categories which persisted after adjustment for multiple hypothesis testing. Interestingly, each of these seven significant correlations was negative; i.e., lower (prenatally more androgenized) normalized male 2D:4D values were cross-nationally related to higher normalized male disease-specific death rates. This agrees with the observed positive association of normalized male 2D:4D with normalized male life expectancy and underlines the reliability of the finding. Although conclusions of these national level findings for the individual level should be drawn very cautiously, the results suggest that lower (prenatally more androgenized) 2D:4D might be sex-specifically associated with lower life expectancy.

In support of our previous individual level observation of lower 2D:4D in males but not in females who have died from suicide in comparison to sex-specific controls with other causes of death (Lenz et al. 2016), we found, in this study, that the normalized male 2D:4D correlated negatively with the normalized male suicide rates (confirmatory hypothesis) and deaths from intentional injuries and self-harm (statistical trend in the exploratory analysis). These findings are also consistent with individual level studies, showing that 2D:4D is involved in aggression-related injuries (Joyce et al. 2013). They indicate that lower (prenatally more androgenized) 2D:4D might be sex-specifically linked to increased risk for suicide, intentional injuries, and self-harm. This finding is relevant, because suicides represent the second leading cause of death among adolescents and young adults 15–29 years of age (WHO 2014b). It might be hypothesized that depression mediates the association between lower 2D:4D and death from suicide. However, the relevance of this mechanism needs further investigation because of inconsistent literature pointing to an increased risk for depression in individuals with both higher 2D:4D (Bailey and Hurd 2005; Smedley et al. 2014) and lower 2D:4D (Martin et al. 1999; Rapoza 2017).

It must be noted that life expectancy and suicidal behaviors are also influenced by a lot of other biological and non-biological factors. Thus, it is unlikely that 2D:4D alone might serve as an accurate predictor of life expectancy and death from suicide in the future.

To our knowledge, this is the first hypothesis-free investigation which shows after adjustment for multiple hypothesis testing significant cross-national correlations between normalized male 2D:4D and normalized male death rates from respiratory infections, asthma, and other respiratory diseases. On an individual level, these findings indicate that lower (prenatally more androgenized) 2D:4D might be sex-specifically related to deaths caused by respiratory infections and asthma. The importance of this observation is underlined by the fact that lower respiratory infections take rank 3 (3.2 million global deaths) on the 2015 WHO top 10 list of global causes of death (WHO 2017). It is tempting to speculate that prenatal sex hormone effects on adolescent and adult smoking behavior may mediate the association of 2D:4D with respiratory infections and asthma. However, this mechanism is improbable, because higher 2D:4D has been found in smokers in comparison to non-smokers (Manning and Fink 2011; Borkowska and Pawlowski 2013) and because 2D:4D has been positively correlated with the severity of nicotine dependence in male alcohol-dependent smokers and ex-smokers (Lenz et al. 2017). Consistent with the concept of dysanapsis, i.e., an incongruent growth of the lungs and airways, it is more likely that early in life-induced direct pulmonary androgenization entails a higher risk for asthma and respiratory infections in later life; e.g., the female fetal lungs start to produce surfactant earlier and female newborns have lower specific airway resistance. Accordingly, boys are two to four times more prone to asthma than girls; however, after puberty, females are at higher risk for asthma, thereby suggesting that pubertal and postpubertal effects are also very important (Townsend et al. 2012).

The exploratory analyses show FDR-adjusted negative cross-national correlations of normalized male 2D:4D with normalized male death rates from neurological conditions and dementias. These results agree with a previously reported sex-specific role of 2D:4D in Alzheimer’s disease based on individual data (Vladeanu et al. 2014). The fact that Alzheimer’s disease and other dementias take rank 7 (1.5 million deaths) on the 2015 WHO top 10 list of global causes of death (WHO 2017) highlights the finding’s relevance. Furthermore, the exploratory analyses revealed that the normalized male 2D:4D values are related to the normalized male death rates of ischemic heart diseases (nominally significant), tuberculosis, and stroke (trends of significance). On the 2015 WHO top 10 list of global causes of death, these illnesses rank at 1, 2, and 9. The observed positive cross-national association between normalized male 2D:4D and normalized male death rates in ischemic heart disease fits with the previous reports of higher 2D:4D values in males with coronary heart disease (Lu et al. 2015) and in males (but not females) with myocardial infarction (Kyriakidis et al. 2010).

The major strength of this investigation is that in addition to verifying the confirmatory hypotheses, we applied a hypothesis-free approach and found significant results after statistical adjustment for multiple hypothesis testing. Moreover, it is important to note that the national 2D:4D values analyzed here represent a large study cohort with a broad age range [> 99% between 10 and 70 years of age (Reimers 2007)].

There are several important limitations. The number of 23 countries investigated is relatively small. The self-measure method applied to quantify 2D:4D reduces the precision. Self-measured 2D:4D show an estimated reliability of 46% of that of expert-measured 2D:4D (Hönekopp and Watson 2010). These factors may be reasons why we here failed to statistically verify associations with lower effect sizes. They may also explain why we were not able to provide further empirical support of previously described significant associations of 2D:4D with alcohol dependence (Kornhuber et al. 2011; Han et al. 2016; Lenz et al. 2017) or eating disorders (Quinton et al. 2011). The analytical approach is limited in that it is based on national gender variations and, thus, depends on a sex-specific relationship between 2D:4D and variable of interest. Moreover, it is important to mention that we explicitly focused on deaths as the endpoint of disease. It may be that 2D:4D is associated with disease prevalence (not in the scope of this study) although not with related mortality. Another important limitation is that our findings cannot easily be generalized to non-Caucasian ethnicities or middle or low income economies, due to the sample of countries that have been included in this analysis. The methodological approach to correlate normalized sex-specific 2D:4D with normalized sex-specific death rates across nations did not allow for the investigation of the role of 2D:4D in sex-specific illnesses such as prostate cancer. Although the reported cross-national correlations of normalized sex-specific 2D:4D with normalized sex-specific death risk from suicide, intentional injuries, and ischemic heart disease agree with the previous reports based on data from individual participants, it remains to be seen to what extent findings such as the national association between 2D:4D and respiratory diseases are relevant to individuals. It is very important to mention that our conclusions drawn from the national level might not correctly reflect true associations on the individual level. The limitations claim a very cautious interpretation of the results reported; an independent replication and support on the individual level, in addition to what is demonstrated here on the national level, is certainly needed. Finally, the limitations related to the use of 2D:4D as a proxy should not be forgotten (e.g., other influencing factors aside from prenatal sex hormone exposure).

## Conclusions

We provide novel national level evidence for a sex-specific role of prenatal sex hormone priming in life expectancy and possible causes of death, such as suicide and neurological, pulmonary, and cardiovascular diseases. The results may yield new insight into the mechanisms underlying lethal disease and build a basis to establish predictive and preventive strategies in the future.

## List of abbreviations

2D:4D: Second-to-fourth finger length ratio
FDR: False discovery rate
WHO: World Health Organization
